# Hepatoprotective Effects of Different Extracts From *Triphala* Against CCl_4_-Induced Acute Liver Injury in Mice

**DOI:** 10.3389/fphar.2021.664607

**Published:** 2021-07-05

**Authors:** Xichuan Wei, Chuanhong Luo, Yanan He, Haozhou Huang, Fei Ran, Wei Liao, Peng Tan, Sanhu Fan, Yuan Cheng, Dingkun Zhang, Junzhi Lin, Li Han

**Affiliations:** ^1^ State Key Laboratory of Southwestern Chinese Medicine Resources, Pharmacy School, Chengdu University of Traditional Chinese Medicine, Chengdu, China; ^2^ Sichuan Academy of Traditional Chinese Medicine, State Key Laboratory of Quality Evaluation of Traditional Chinese Medicine, Chengdu, China; ^3^ Sanajon Pharmaceutical Group, Chengdu, China; ^4^ TCM Regulating Metabolic Diseases Key Laboratory of Sichuan Province, Hospital of Chengdu University of Traditional Chinese Medicine, Chengdu, China

**Keywords:** *Triphala*, extraction process, CCl4-induced acute liver injury, Nrf-2 signaling pathway, hepatoprotective effects, bioactivity consistency

## Abstract

**Background:** *Triphala* is a traditional polyherbal formula used in Indian Ayurvedic and Chinese Tibetan medicine. A wide range of biological activities have been attributed to *Triphala*, but the impact of various extraction methods on efficacy has not been determined.

**Purpose:** The study aimed to evaluate *Triphala* extracts obtained by various methods for their hepatoprotective effects and molecular mechanisms in a mouse model of carbon tetrachloride (CCl_4_)-induced liver injury.

**Methods:** HPLC fingerprinting was used to characterize the chemical characteristics of *Triphala* extracts obtained by (a) 0.5 h ultrasonication, (b) 2 h reflux, and (c) 4 h reflux. Hepatoprotective efficacy was evaluated in a mouse model of CCl_4_-induced liver damage. Serum levels of alanine transaminase (ALT) and aspartate aminotransferase (AST) were measured, as well as the liver antioxidant and inflammatory markers malondialdehyde superoxide dismutase glutathione peroxidase (GSH-Px), TNF-α, and IL-6. Gene and protein expression of Nrf-2 signaling components Nrf-2, heme oxygenase (HO-1), and NADPH Quinone oxidoreductase (NQO-1) in liver tissue were evaluated by real-time PCR and western blotting.

**Results:** Chemical analysis showed a clear difference in content between extracts produced by ultrasonic and reflux methods. The pharmacological analysis showed that all three Triphala extracts reduced ALT, AST, MDA, TNF-α, and IL-6 levels and increased SOD and GSH-Px. Triphala extracts also induced transcript and protein expression of Nrf-2, HO-1, and NQO-1.

**Conclusion:** Triphala extract prevents CCl_4_-induced acute liver injury. The ultrasonic extract of Triphala was most effective, suggesting that hepatoprotection may be related to the larger tannins via activation of Nrf-2 signaling.

## Introduction

As one of the largest metabolic organs in the human digestive system, the liver has functions of transformation, excretion, immunity, and detoxification and is susceptible to chemical liver injury caused by various pathogenic and stimulating factors. Aggravated liver cell damage, if left unchecked, can lead to hepatitis, liver fibrosis, or irreversible cirrhosis, which eventually leads to liver cancer (Gu and Manautou, 2012). Studies have shown that the primary pathogenic mechanism of chemical liver injury is the oxidative stress caused by the accumulation of reactive oxygen radicals in the liver and the resulting inflammatory response (Souza et al., 2018).

*Triphala* is a traditional polyherbal medicine comprised of *Terminalia chebula* Retz., *Terminalia bellirica* (Gaertn.) Roxb.., and *Phyllanthus emblica* Linn., each of which contains a variety of chemical substances with biological activity (Baliga et al., 2012; Zhao et al., 2015; Chen et al., 2019; Nigam et al., 2020). *Triphala* has a long history in Indian and Chinese traditional medicine as a complementary and alternative therapy for chronic diseases (Jaiswal et al., 2016; Prasad and Srivastava, 2020). *Triphala* is considered a multipurpose therapeutic drug with anti-inflammatory, analgesic, hypoglycemic, antibacterial, and antioxidant properties (Srikumar et al., 2006; Rasool and Sabina, 2007; Peterson et al., 2017; Luo et al., 2019). In traditional usage, *Triphala* is applied in the treatment of gastritis, hepatitis, colitis and other digestive diseases (Deep et al., 2005; Li et al., 2018a; Nariya et al., 2011; Olennikov et al., 2015; Rayudu and Raju, 2014; Wang et al., 2018). *Triphala* also has potential uses in the treatment of obesity and diabetes, as well as retinopathy and cardiovascular and cerebrovascular diseases (Saravanan et al., 2007; Gurjar et al., 2012; Kamali et al., 2012; Lu et al., 2012; Ganeshpurkar et al., 2015). Although *Triphala* is widely used throughout Asia, clinical safety data are lacking. In Thailand, a phase I clinical observational trial was performed in 20 healthy volunteers (10 male, 10 female) to verify the safety of *Triphala*. In that study, a water extract of *Triphala* had no obvious side effects (Phetkate et al., 2020). Pharmaceutical analyses have shown that *Triphala* is rich in saponins, terpenes, tannins, flavonoids, and phenolic acids (Avula et al., 2013). The hydrolyzed tannins in *Triphala* are considered the primary inducers of biological activity (Pawar et al., 2009; Russell et al., 2011).

Tannins are known to provide hepatoprotection (Olennikov et al., 2015). There are various research models for inducing liver injury in which *Triphala* and related plant medicinals have been tested for hepatoprotective activity. However, the preparation methods used in these studies vary and may confound the results (Table 1). In a previous study Huang et al. (2019), we found that the *Phyllanthus emblica* component of *Triphala* exhibited hydrolytic tannin conversion in heat- and reflux-mediated extraction, suggesting various extraction methods may yield extracts with variable biological activities. Published studies include the use of common hot and cold extraction methods for *Triphala* and related plants, but extraction temperature influences the chemical composition of *Triphala*.

**TABLE 1:** Preparation methods for *Triphala* and related botanical medicines.

Sample	Liver injury model	Preparation of plant extract	References
*Terminalia chebula* fruit	Diazinon-induced hepatotoxicity	*Chebula* fruits were air-dried at room temperature, then ground and extracted with ethanol and water (70:30, v/v)	Ahmadi-Naji et al. (2017)
*Emblica officinalis* fruit	Ochratoxin-induced lipid peroxidation in the kidney and liver	Dried fruits were ground to a powder, then mixed in distilled water (5 g in 100 ml) and mixed for 3 h at 40°C	Chakraborty and Verma (2010)
*Phyllanthus emblica* L. bark	Ethanol-induced hepatotoxicity	Dried bark powder (100 g) was extracted in 250 ml of a 7:3 mixture of absolute ethanol and water. In a rotary evaporator, the extract was evaporated to a dry state by vacuum distillation	Chaphalkar et al. (2017)
*Terminalia chebula*	t-BHP- induced acute liver injury	Samples (100 g) were extracted in 1 L distilled water, boiled for 90 min, centrifuged for 15 min, and the supernatant lyophilized	Choi et al. (2015)
*Phyllanthus emblica* L. fruit	High fat diet-induced liver injury	The dried powder was extracted with water using a rotary shaker at room temperature for 24 h and dried by vacuum evaporation	Huang et al. (2017)
*Terminalia bellirica* (Gaertn) Roxb. Fruit	CCl_4_-induced hepatotoxicity	Dried fruits were minced and extracted with ethanol, then dried under reduced pressure	Jadon et al. (2007)
*Emblica officinalis* fruit	Iron dextran-induced l hepatotoxicity	A mixture of 100 g powder and 500 ml methanol: Water (7:3) was stirred with a magnetic stirrer for 15 h, then the mixture was centrifuged. The supernatant was collected, concentrated in a rotary evaporator, and freeze-dried	Sarkar et al. (2015)
*Terminalia bellirica* and *Terminalia sericea* leaf	d-galactosamine-induced liver damage	Leaves were air-dried, ground, and extracted with methanol at room temperature for 3 days, then freeze-dried	Sobeh et al. (2019)
*Triphala*	DMH-induced liver damage	*Triphala* was mixed at 5% w/w with diet and pressed into pellets	Sharma and Sharma (2011)
*Terminalia chebula* fruit	Young and aged rats	Dried peels were placed in 800 ml distilled water and heated in a water bath at 40°C for 24 h	Mahesh et al. (2009)
*Phyllanthus*	CCl_4_-induced hepatotoxicity	Powder was dissolved in 250 ml methanol followed by soxhlet extraction at 80°C for 8 h, filtration, and concentration under reduced pressure	Lee et al. (2006)
*Phyllanthus emblica* L	Paracetamol, CCl_4_, ethanol-induced hepatic damage	Hepatoprotective herbal tablets were prepared by direct compression	Tatiya et al. (2012)
*Padma hepaten*	t-BHP-induced oxidative hepatotoxicity in cultured rat hepatocytes	Padma hepaten (50 mg) in 60% methanol (4 ml), extracted by ultrasonication for 30 min 40°C	Olennikov et al. (2015)
*Terminalia bellirica* (Gaertn) Roxb. Fruit	Acute toxicity with aqueous acetone extract	Powder (100 g) was degreased with petroleum ether, suspended in 70% acetone in water (300 ml), extracted with a mechanical shaker for 72 h, concentrated in a rotary evaporator, and lyophilized	Jayesh et al. (2017)
*Phyllanthus emblica* L. leaf	Diethyl nitrosamine--induced hepatocellular carcinoma	Powder (10 g) mixed in 100 ml double distilled water and heated in a 70°C water bath for 30 min	Singh et al. (2019)
*Phyllanthus emblica* L	Isoniazid, rifampicin, and pyrazinamide-induced hepatic damage	Powder (10 g) mixed in 40 ml distilled water and heated for 2 h	Panchabhai et al. (2008)
*Triphala*	Paracetamol-induced hepato-renal toxicity	*Triphala* soaked overnight in distilled water, filtered and concentrated in a rotary evaporator, then freeze-dried under vacuum for 50 h	Singh and Mani (2015)
*Phyllanthus emblica* L. leaf	Arsenic-mediated toxicity	250 g of dried leaf powder was suspended in 1 L of 95% ethanol and dried powdered leaves (250 g) suspended in 95% ethanol (1 L) and extracted at room temperature for 10 days, filtered, and concentrated in a rotary evaporator under vacuum	Sayed et al. (2015)
*Terminalia bellirica* (Gaertn.) Roxb. Fruit	CCl_4_-induced hepatotoxicity	Powdered fruit was extracted in a mechanical shaker with 70% aqueous acetone for 72 h. After the solvent was completely evaporated, the extract was filtered and lyophilized	Jayesh et al. (2019)
*Terminalia chebula* (Retz.) fruit	Iron-induced hepatotoxicity	Use a magnetic stirrer to stir the powder with methanol: Water (7:3) for 15 h, and then centrifuge the mixture. The extract was filtered, evaporated, and lyophilized	Sarkar et al. (2012)
*Terminalia bellirica* (Gaertn.) Roxb. Fruit	CCl_4_-induced hepatotoxicity	The powder was extracted with an aqueous acetone solution in a mechanical shaker for 72 h. The extract was filtered, evaporated, and lyophilized	Kuriakose et al. (2017)

In this study, we compared the hepatoprotective efficacy of *Triphala* extracts obtained by various methods. We established a mouse model of carbon tetrachloride (CCl_4_)-induced liver damage, then monitored for the presence of oxidative damage markers alanine transaminase (ALT) and aspartate aminotransferase (AST), as well as the liver antioxidant and inflammatory markers malondialdehyde (MDA), superoxide dismutase (SOD), glutathione peroxidase (GSH-Px), as well as inflammatory factors and Nrf2 signaling-related genes and proteins to explore the protective effect and mechanism of the various *Triphala* extracts. The results of this study will support future clinical applications of *Triphala*.

## Materials and Methods

### Materials and Reagents

*Terminalia chebula* Retz., *Terminalia bellirica* (Gaertn.) Roxb. and *Phyllanthus emblica* Linn. were purchased from Zhongyong Pharmaceutical Co., Ltd. (Sichuan, China). All herbs were identified by Professor Jin Pei, deposited at the Chengdu University of TCM, and met Chinese Pharmacopoeia requirements (2015 Edition). Standards of Chebulic acid (CHB180831), Gallic acid (CHB171107), Punicalin (CHB190211), Catechin (CHB170301), Epigallocatechin gallate (CHB180307), Epicatechin (CHB180831), Corilagin (CHB190106), Gallocatechin gallate (CHB180327), 1,3,6-tri-O-galloylglucose (CHB191021), Epicatechin gallate (CHB170317), Ferulic acid (CHB 180201), Chebulagic acid (CHB190109), 1,2,3,4,6-O-penta-galloyl glucose (CHB190125), Chebulinic acid (CHB190124), and Ellagic acid (CHB170303) were purchased from Chengdu Chroma-Biotechnology Co., Ltd. (Chengdu, China), the purity of all standard products is ≥ 98%. CCl_4_ (20181010) was purchased from Tianjin Bodi Chemical Co., Ltd. (Tianjin, China), Dimethyl diphenyl bicarboxylate (DDB) (200603) was purchased from Bond Pharmaceuticals Group Co., Ltd. (Wenling, China). Test kits for ALT (20191003), AST (20191005), SOD (20191101), MDA (20191028), and GSH-Px (20191029) were obtained from Nanjing Jiancheng Bioengineering Institute (Nanjing, China). TNF-α (A28291045) and IL-6 (A20691132) were obtained by Multi Sciences Biotech Co., Ltd. (Hangzhou, China).

### Sample Preparation

Plant materials were prepared in traditional proportions (3 *Terminalia chebula*: 2 *Terminalia bellirica*: 2.4 *Phyllanthus emblica*). Triphala is extracted in water solvent and extracted by ultrasonication for 0.5 h (U-0.5 h) at 20°C, reflux for 2 h (R-2 h) at 100°C, and reflux for 4 h (R-4 h) at 100°C. The solution concentration was 0.24 g/ml, and the extraction values of the three methods were 12.74%, 34.91%, and 41.30%, respectively. The extracts were filtered, and the filtrates were stored in a refrigerator at 4°C.

### Experimental Animals

Kunming mice (30 ± 2 g) were supplied by Dashuo Laboratory Animal Co. Ltd. (Chengdu, China). The animals were housed at room temperature under a 12:12 light:dark schedule with food and water ad libitum. All experiments were performed in strict accordance with the recommendations of China’s “Guidelines for the Care and Use of Laboratory Animals.” The experimental protocol was approved by the Ethics Committee of the Affiliated Hospital of Chengdu University of TCM (Approval ID: 2018BL-002).

The animals were divided into nine groups, with an average of six mice per group. The experimental groups received one of the three Triphala extracts (U-0.5, R-2, and R-4 h), with treatments administered by gavage at 1.2 g/kg for the low-dose group (L) and 2.4 g/kg for the high-dose group (H). Dosages were calculated based on the clinical dosage of Triphala by the body surface area method. Positive controls received DDB (7.5 mg/kg) by gavage. Normal (N) and Model (M) controls were given the same volume of distilled water by gavage. All groups were treated intragastrically once daily for a week. Two hours after treatment on the last day, all mice but those in the N group were given 0.1% CCl_4_ vegetable oil solution (10 ml/kg body weight) by intraperitoneal injection, while the mice in the group N were merely given the same amount of vegetable oil. All animals were fasted overnight and sacrificed after 16 h. Blood and liver tissues were collected immediately. The collected blood was centrifuged at 4,000 rpm at 4°C for 10 min and stored at –20°C. The liver tissues were dissected and immediately rinsed with ice-cold saline. One portion was immediately refrigerated at –80°C, and the other was fixed with 4% paraformaldehyde for histopathological analysis.

### HPLC Conditions and Analysis

The sample concentration is too high for liquid chromatography analysis, so samples were diluted 10-fold and analyzed by Shimadzu LC-20AT HPLC (Shimadzu Corporation, Kyoto, Japan) on Welchrom C_18_ columns (4.6 × 250 mm, 5 μm; Shanghai Yuexu Material Technology Co., Ltd., China). Detection conditions were as follows: wavelength 270 nm, column temperature was 25°C, mobile phase flow rate 1 ml min^−1^, injection volume 10 μL. The mobile phase was 0.2% aqueous phosphoric acid and methanol, adopting a gradient elution program of 5% of B at 0–6 min, 5%–7% of B at 6–15 min, 7%–15% of B at 15–20 min, 15%–21% of B at 20–25 min, 21%–22% of B at 25–41 min, 22%–28% of B at 41–47 min, 28%–32% of B at 47–55 min, 32%–37% of B at 55–61 min, 37%–38% of B at 61–62 min, 38%–39% of B at 62–67 min, 39%–45% of B at 67–70 min, 45%–65% of B at 70–80 min, 65%–5% of B at 80–90 min (Huang et al., 2018).

### UPLC-Q-Orbitrap HRMS Conditions and Analysis

The analysis was performed on ultra-high performance liquid chromatography coupled with quadrupole-orbitrap high resolution mass (UPLC-Q-Orbitrap HRMS) (Thermo Fisher, United States). Chromatographic separation was carried out at 30°C on Thermo Scientific Accucore C_18_ (2.1 mm × 100 mm, 2.6 μm). The mobile phase consisted of (A) water with 0.1% formic acid and (B) methanol. The gradient was as follows: 0–25 min, 5% B isocratic; 25–30 min, 5–95% B linear; 30–35 min, 50% B isocratic. The flow rate was 0.3 ml/min. The MS acquisition was performed using both positive and negative ionization mode. The heated electrospray ionization parameters as follows: sheath gas flow 35 arb (arbitrary units), auxiliary gas flow 10 arb, spray voltage 3.0 kV for positive ionization and negative ionization, capillary temperature 320°C, probe heater temperature 350°C, the ion scanning range is m/z 100–1500.

Under the above conditions, the chemical constituents of the three extracts of Triphala were qualitatively analyzed. Use Xcalibur 3.0 software to process the total ion chromatogram of the sample in positive and negative ion mode, match the measured spectrum with the mzCloud and mzVault network databases, and then combine the precise relative molecular mass of the target component, reference substances, MassBank, and Human Metabolome database (HMDB), PubMed, ChemSpider and related references, for manual identification and identification.

### Calculation of Body Weight and Liver Index

Every two days throughout the experiment, the mice were weighed, and changes were noted. Dissected mouse livers were weighed, and the liver index was calculated as liver index = liver mass (g)/mouse body weight (g) × 100%.

### Biochemical Examinations of Serum ALT, AST Levels

Serum levels of ALT and AST were determined using standard kits according to the manufacturer’s instructions.

### Histopathology

The liver tissue stored in 4% paraformaldehyde solution at 4°C was embedded in paraffin, then sliced by microtome, stained by hematoxylin and eosin (H&E), and finally examined under an optical microscope (Nikon eclipse ci, Japan).

### Measurement of Hepatic MDA, GSH-Px, SOD, TNF-α and IL-6 Levels

Hepatic levels of MDA, SOD, GSH-Px, TNF-α, and IL-6 were determined according to the manufacturer’s instructions of standard assay kits.

### RNA Extraction, Reverse Transcription-PCR, and RT-PCR

Total RNA was extracted from mouse liver using Trizol (Invitrogen Life Technologies), followed by reverse transcription to cDNA (Applied Biological Materials Inc.) and PCR amplification (Shanghai Hongshi Medical Technology Co., Ltd.). Amplification conditions were: initial denaturation at 95°C for 10 min, followed by 40 cycles of denaturation at 95 °C for 15 s, annealing at 60°C for 15 s, and extension for 60 s. Amplification targets were the Nrf-2, NQO-1, and HO-1 genes, with primers described in Supplementary Table S1. Relative expression was determined by the 2^−ΔΔCT^ method.

### Western Blot Analysis

Liver tissue was weighed, washed 2–3 times with ice-cold PBS, mixed with a 10-fold volume of RIPA, homogenized on ice, centrifuged at 12,000 *g* for 10 min, and the supernatant collected. Protein concentration was determined by the BCA method. Total protein (15 μL) was separated by 10% SDS-PAGE, then transferred to a PVDF membrane (Millipore Corporation, United States) overnight at 25 V. The membrane was sealed in 5% defatted milk/TBST for 1 h and incubated with primary antibody overnight at 4°C. The membrane was rinsed three times with TBST at room temperature, incubated with secondary antibody at room temperature for 30 min, then washed three times with TBST. Protein bands were detected by ECL, and the images were collected with a chemiluminescence imaging system (Shanghai Clinx Scientific Instrument Co., Ltd.). Grayscale analysis was performed with the on-instrument ChemiScope software.

### Statistical Analysis

Data analysis was performed using SPSS 21.0 statistical analysis software with results expressed as mean ± standard deviation. Analysis of variance (ANOVA) and LSD *t*-tests were used to compare multiple groups. Significance was defined as *p <* 0.05.

## Results

### Chemical Analysis

**FIGURE 1:**
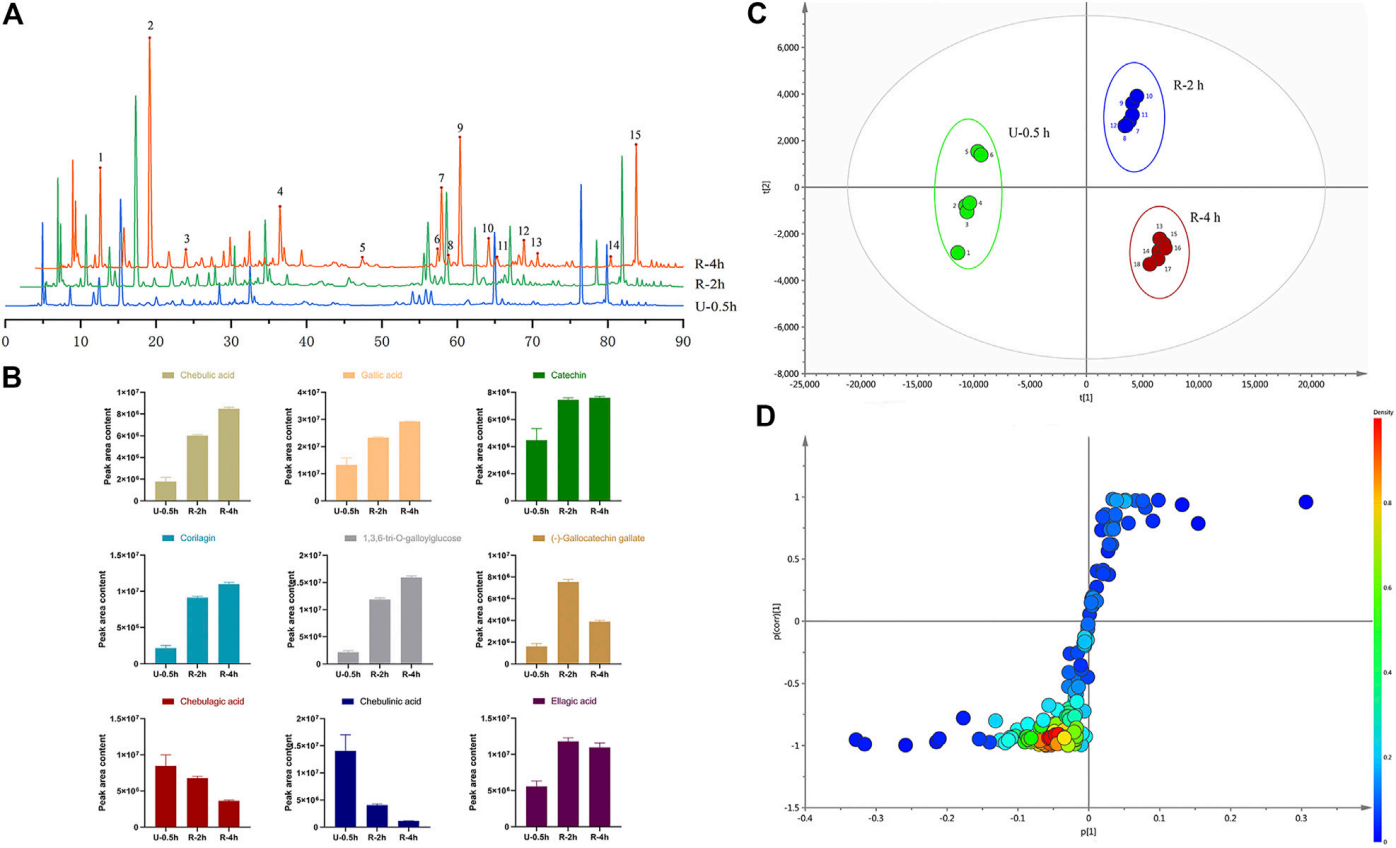
**(A)** HPLC of *Triphala* in different extraction process (1. Chebulic acid, 2. Gallic acid, 3. Punicalin 4. Catechin, 5. Epigallocatechin gallate, 6. Epicatechin, 7. Corilagin, 8. Gallocatechin gallate, 9.1,3,6-tri-O-galloylglucose, 10. Epicatechin gallate, 11. Ferulic acid, 12. Chebulagic acid, 13. 1,2,3,4,6-penta-O-galloyl glucose, 14. Chebulinic acid, 15. Ellagic acid). **(B)** The variation of peak area of composition in different decoction time. **(C)** PCA score of extracts of *Triphala*. **(D)** S-plot of extracts of *Triphala*.

HPLC chromatograms are shown in Figure 1A. Through the comparison of reference substance, we carried out quantitative analysis of 15 components, as shown in Table 2. We compared the differences of 9 components with large changes in peak area during the decoction of *Triphala* (Figure 1B). Multivariate statistical methods were used to analyze the fingerprint data, and SIMCA-P 13.0 software was used to perform PCA analysis. The PCA score chart shows differences between the products of the three extraction methods, particularly between the ultrasonic and reflux methods (Figure 1C). Orthogonal projections to latent structures discriminant (OPLS-DA) and S-plot analysis were used to find differences in chemical markers. The S-plot is a loading profile that depicts the influence of variables on biomarker selection. Among all the 186 variables in the S-plot, we identified 10 chemical markers that differed most between the *Triphala* preparations (Figure 1D). The retention times for these markers were 15.230, 56.484, 54.018, 8.614, 79.821, 60.271, 32.466, 12.475, 64.938, and 76.437. By comparison with the reference substance’s retention time, nine of the components were determined to be gallic acid, 1,3,6-tri-O-galloylglucose, corilagin, chebulic acid, ellagic acid, epicatechin gallate, catechin, chebulagic acid, and chebulinic acid. These 9 identified actives have a wide range of biological activities (Table 3). The component most reduced by extraction is hydrolyzed tannin (chebulinic and chebulagic acid), while tannin hydrolysate was greatly increased (corilagin, gallic acid, chebulic acid, and ellagic acid).

**TABLE 2:** The variation of composition content in different decoction time ($\bar{x}$ ± s,*n* = 6).

Chemical name	U-30 min (mg/ml)	R-2 h (mg/ml)	R-4 h (mg/ml)
Chebulic acid	0.3935 ± 0.08	1.3230 ± 0.02	1.8656 ± 0.03
Gallic acid	0.4051 ± 0.08	0.7128 ± 0.01	0.8908 ± 0.01
Punicalin	0.1329 ± 0.02	0.2287 ± 0.01	0.2213 ± 0.01
Catechin	0.3907 ± 0.07	0.6497 ± 0.01	0.6624 ± 0.01
Epigallocatechin gallate	0.0933 ± 0.02	0.1995 ± 0.01	0.1988 ± 0.01
Epicatechin	0.1454 ± 0.03	0.7436 ± 0.02	0.4167 ± 0.02
Corilagin	0.1558 ± 0.03	0.6542 ± 0.01	0.7878 ± 0.02
Gallocatechin gallate	0.0436 ± 0.01	0.0461 ± 0.00	0.0500 ± 0.00
1,3,6-tri-O-galloylglucose	0.1452 ± 0.02	0.7998 ± 0.02	1.0721 ± 0.02
Epicatechin gallate	0.0978 ± 0.02	0.4568 ± 0.02	0.2354 ± 0.01
Ferulic acid	0.3635 ± 0.09	3.4968 ± 0.10	3.3463 ± 0.06
Chebulagic acid	0.6127 ± 0.11	0.4902 ± 0.02	0.2627 ± 0.01
1,2,3,4,6-penta-O-galloylglucose	0.0201 ± 0.00	0.0790 ± 0.00	0.0751 ± 0.01
Chebulinic acid	0.9132 ± 0.19	0.2643 ± 0.01	0.0736 ± 0.00
Ellagic acid	0.0889 ± 0.01	0.1882 ± 0.01	0.1748 ± 0.01

**TABLE 3:** Pharmacological activities of 9 identified actives.

Actives name	Pharmacological activity	References
Gallic acid	Antioxidant, antimicrobial, anti-carcinogenic, anti-inflammatory etc.	Choubey et al. (2018)
Chebulic acid	Antioxidant, anti-fibrotic, anti-inflammatory, antiglycative etc.	Yoo et al. (2020)
1,3,6-tri-O-galloylglucose	Hemostatic, anti-inflammatory, and antiviral bioactivities	Gong et al. (2020)
Corilagin	Antioxidant, anti-tumor, hepatoprotective, and anti-inflammatory	Li et al. (2018a), Li et al. (2018b)
Ellagic acid	Antioxidant, anti-inflammatory, hepatoprotective, anti-diabetic	Derosa et al. (2016)
Epicatechin gallate	Antioxidant, anti-tumor	Fu et al. (2019)
Catechin	Anticancer, anti-obesity, anti-inflammatory, and antioxidant	Nakano et al. (2019)
Chebulagic acid	Antioxidant, anti-inflammatory, anti-proliferative, anti-tuberculosis, antiviral, neuroprotective, anti-thrombotic	Lu et al. (2019)
Chebulinic acid	Anti-oxidant, anti-cancer, antihypertensive activities etc.	Munawar et al. (2019)

**FIGURE 2:**
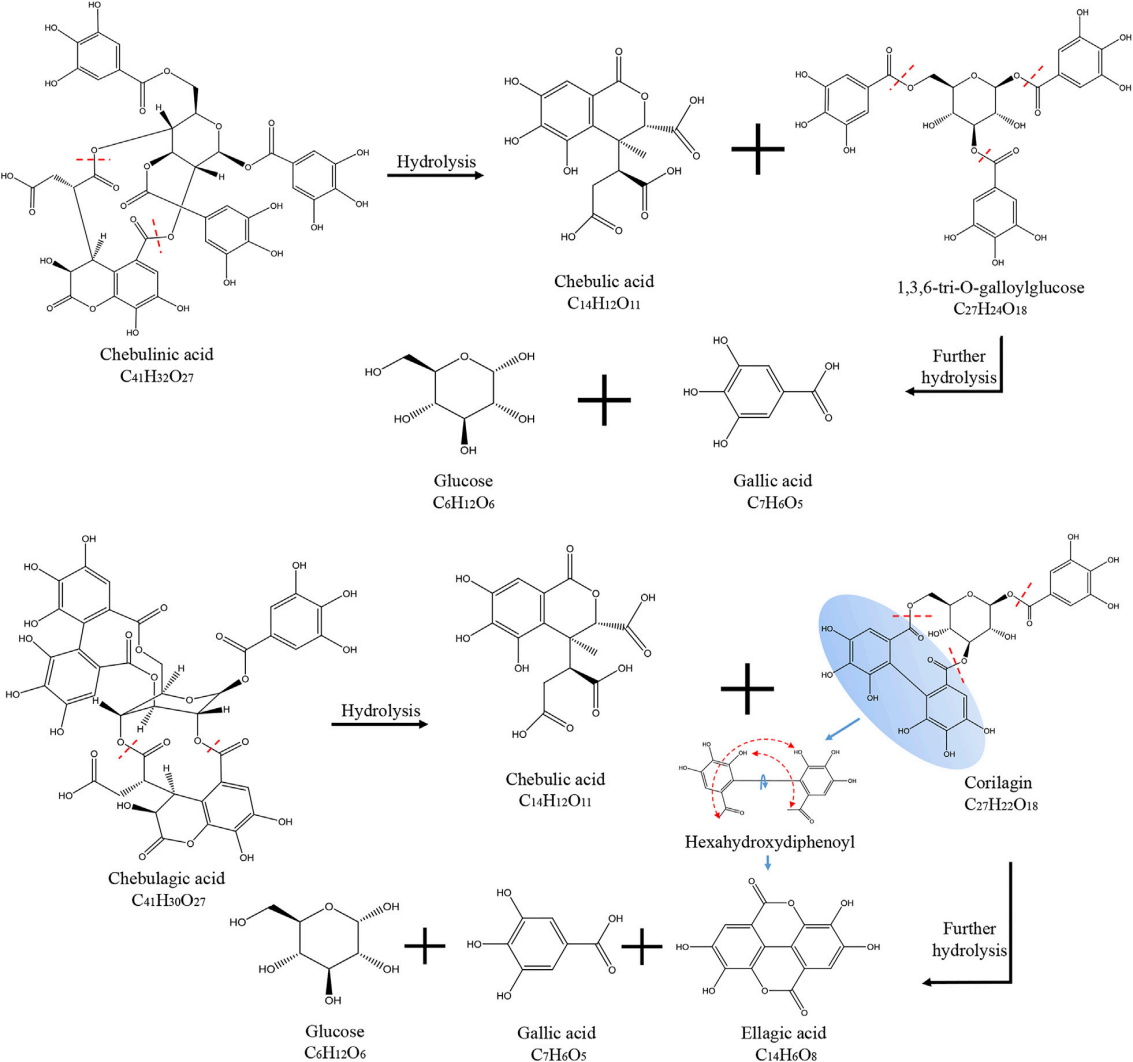
Schematic diagram of the hydrolysis of Chebulinic acid and Chebulagic acid.

Chebulinic acid decreased most dramatically during the decoction of Triphala, likely due to its chemical structure. Chebulinic acid is formed by the condensation of small molecule tannins and glucose through five ester bonds. It is not stable under heat and easily decomposes into one molecule of chebulic acid and one molecule of 1,3,6-Tri-O-galloyl glucose, which is then decomposed into three molecules of gallic acid and one molecule of glucose. Chebulagic acid is also unstable under heated conditions and is hydrolyzed to produce one molecule of chebulic acid and one molecule of corilagin. Corilagin is composed of a galloyl, a hexahydroxybiphenoyl (HHDP) and a glucosyl moiety by three ester bonds. When heated, it will continue to hydrolyze to produce gallic acid, ellagic acid, and glucose. A schematic diagram of the hydrolysis of chebulinic and chebulagic acid is shown in Figure 2. *Triphala* also contains catechins, condensed tannins that undergo condensation reactions and precipitate, thus reducing the final extract’s content.

Through the UPLC-Q-Orbitrap HRMS, a total of 106 compounds were identified in the three extracts of *Triphala*, among which 76 were common components, as shown in Table 4. The results showed that *Triphala* mainly contained tannins, phenolic acids, flavonoids, alkaloid sugars and glycosides, vitamins, amino acids, fatty acids, organic acids, and a small amount of coumarins, terpenoids, sterols, lignin.

**TABLE 4:** UPLC-Q-Orbitrap HRMS identification results.

Peak NO	tR/min	Name	Origin	Formula	Theoretical [M-H]^−^/[M + H]^+^	Measured [M-H]^-^/[M + H]^+^	δ/ppm	Primary fragment ion m/z
1	1.06	Mannitol	1.2.3	C_6_H_14_O_6_	181.0712/	181.0712/	0.00	71.0128, 101.0234
2	1.08	d-fructose	1.2.3	C_6_H_12_O_6_	179.0556/	179.0555/	−0.56	87.0077, 161.0084
3	1.10	Choline	1.2.3	C_5_H_13_NO	/104.1075	/104.1074	−0.96	60.0814
4	1.12	Betaine	1.2.3	C_5_H_11_NO_2_	/118.0869	/118.0865	−3.39	58.0658, 59.0736
5	1.13	l-Proline	1.2.3	C_5_H_9_NO_2_	/116.0712	/116.0709	−2.58	70.0658
6	1.13	Trigonelline	1.2.3	C_7_H_7_NO_2_	/138.0555	/138.0549	−4.35	92.0499, 94.0656
7	1.15	Dulcitol	1.2.3	C_6_H_14_O_6_	181.0712/	181.0709/	−1.66	71.0128, 89.0234, 101.0234
8	1.16	Cheublic acid	1.2.3	C_14_H_12_O_11_	355.0302/	355.0316/	3.94	337.0204, 115.0043
9	1.18	Maleic acid	2.3	C_4_H_4_O_4_	115.0031/	115.0026/	−4.35	71.0128
10	1.20	Malic acid	1.2.3	C_4_H_6_O_5_	133.0137/	133.0133/	−3.01	71.0128, 115.0027, 133.0134
11	1.23	Shikimic acid	1.2.3	C_7_H_10_O_5_	173.0450/	173.0449/	−0.58	93.0337, 111.0443, 137.0237
12	1.38	l-glutathione	1	C_10_H_17_N_3_O_6_S	/309.0916	/309.0912	−1.29	76.0221, 162.0218
13	1.38	Gallocatechin	1.2.3	C_15_H_14_O_7_	305.0661/	305.0672/	3.61	174.9553
14	1.49	4-Aminophenol	1.2.3	C_6_H_7_NO	/110.0606	/110.0603	−2.73	65.0339, 92.0499
15	1.54	Citric acid	1.2.3	C_6_H_8_O_7_	191.0192/	191.0193/	0.52	111.0078, 147.0286, 173.0081
16	1.54	6-Hydroxynicotinic acid	2.3	C_6_H_5_NO_3_	/140.0348	/140.0343	−3.57	122.0239
17	1.55	l-pyroglutamic acid	1.2.3	C_5_H_7_NO_3_	/130.0504	/130.0501	−2.31	84.0448
18	1.71	Succinic acid	1.2.3	C_4_H_6_O_4_	117.0188/	117.0184/	−3.42	73.0284, 117.0183
19	1.77	2-Hydroxycinnamic acid	1.2.3	C_9_H_8_O_3_	/165.0552	/165.0549	−1.82	123.0441, 147.0440
20	1.77	Phenylacetaldehyde	2.3	C_8_H_8_O	/121.0653	/121.0652	−0.83	93.0703, 103.0545
21	1.87	d-saccharic acid	2.3	C_6_H_10_O_8_	209.0298/	209.0298/	0.00	85.0285, 191.0192
22	1.90	dl-norleucine	1.2.3	C_6_H_13_NO_2_	/132.1024	/132.1021	−2.27	69.0705, 86.0969
23	1.90	Pyrogallol	1.2.3	C_6_H_6_O_3_	/127.0395	/127.0391	−3.15	99.0444, 109.1015
24	2.04	Adenosine	2.3	C_10_H_13_N_5_O_4_	/268.1046	/268.1041	−1.86	136.0617
25	2.13	Gallic acid	1.2.3	C_7_H_6_O_5_	169.0137/	169.0134/	−1.78	79.0181, 97.0285, 125.0235
26	2.13	Methyl gallate	1.2.3	C_8_H_8_O_5_	/185.0450	/185.0445	−2.70	125.0234, 14.0333, 153.0181
27	2.23	Punicalin	1.2.3	C_34_H_22_O_22_	781.0524/	781.0528/	0.51	270.9886, 298.9836, 600.9898
28	2.34	Quinic acid	2.3	C_7_H_12_O_6_	191.0556/	191.0555/	−0.52	85.0285, 127.0392
29	2.75	Brevifolincarboxylic acid	1.2.3	C_13_H_8_O_8_	/293.0297	/293.0293	−1.37	191.0338, 219.0287, 247.0235
30	3.00	4-Hydroxy-6-methyl-2-pyrone	2.3	C_6_H_6_O_3_	/127.0395	/127.0393	−1.57	71.0498, 99.0446, 109.0287
31	3.01	l-Phenylalanine	1.2.3	C_9_H_11_NO_2_	/166.0868	/166.0863	−3.01	103.0545, 120.0808
32	3.68	Protocatechuic acid	1.2.3	C_7_H_6_O_4_	153.0188/	153.0187/	−0.65	108.0281, 109.0285
33	3.93	4-Hydroxyquinoline	1.2.3	C_9_H_7_NO	/146.0606	/146.0602	−2.74	77.0391, 91.0546
34	4.62	d-pantothenic acid	2.3	C_9_H_17_NO_5_	/220.1185	/220.1180	−2.27	90.0554, 184.0966, 202.1071
35	5.26	Caprolactam	1.2.3	C_6_H_11_NO	/114.0919	/114.0916	−2.63	114.0915
36	5.38	4-Hydroxybenzoic acid	1	C_7_H_6_O_3_	137.0239/	137.0238/	−0.73	93.0335, 137.0235
37	5.42	Kynurenic acid	1.2.3	C_10_H_7_NO_3_	/190.0504	/190.0498	−3.16	116.0496, 144.0439, 162.0549
38	6.05	3,4-Di-O-Galloylquinic acid	2.3	C_21_H_20_O_14_	495.0775/	495.0777/	0.40	169.0137, 173.0450, 191.0556
39	6.11	N-Acetyltyramine	1	C_10_H_13_NO_2_	/180.1024	/180.1021	−1.67	103.0546, 121.0650
40	6.20	Lycoperodine I	1.2.3	C_12_H_12_N_2_O_2_	217.0977/	217.0974/	−1.38	144.0806
41	6.37	2-Isopropylmalic acid	1.2.3	C_7_H_12_O_5_	175.0606/	175.0608/	1.14	85.0649, 115.0391, 175.0605
42	6.62	Geraniin	1.2.3	C_41_H_28_O_27_	951.0740/	951.0757/	1.79	300.9992, 463.0525
43	6.73	Gallocatechin gallate	1.2.3	C_22_H_18_O_11_	457.0771/	457.0783/	2.63	169.0126
44	7.14	Ethyl gallate	1.2.3	C_9_H_10_O_5_	197.0450/	197.0461/	5.58	169.0126
45	7.24	Epicatechin gallate	1.2.3	C_22_H_18_O_10_	441.0822/	441.0821/	−0.23	125.0221
46	7.75	1,3,6-Tri-O-Galloylglucose	2.3	C_27_H_24_O_18_	635.0884/	635.0859/	−3.94	169.0136, 211.0246, 465.0683, 483.0772
47	7.76	Corilagin	1.2.3	C_27_H_22_O_18_	633.0728/	633.0728/	0.00	300.9988, 463.0518
48	7.78	Quercetin 3-O-Glucuronide	2.3	C_21_H_18_O_13_	/479.0826	/479.0815	−2.30	301.0002
49	8.18	12:4+3O fatty acyl hexoside	1.2.3	C_18_H_28_O_9_	387.1655/	387.1661/	1.55	101.0235, 207.1024
50	8.32	Syringaldehyde	2.3	C_9_H_10_O_4_	/183.0657	/183.0654	−1.64	77.0391, 95.0495, 123.0441
51	8.39	Ginnalin A	2.3	C_20_H_20_O_13_	467.0826/	467.0835/	1.93	125.0236, 169.0137
52	8.53	Ethyl gallate	1.2.3	C_9_H_10_O_5_	197.0450/	197.0449/	−0.51	125.0234, 169.0134
53	8.55	(2S,3S,4S,5R,6R)-6-(3-benzoyloxy-2-hydroxypropoxy)-3,4,5-trihydroxyoxane-2-carboxylic acid	1.2.3	C_16_H_20_O_10_	371.0978/	371.0981/	0.81	113.0236, 121.0287, 249.0616
54	8.77	P-coumaric acid	1.2.3	C_9_H_8_O_3_	163.0395/	163.0392/	−1.84	119.0494, 163.0394
55	8.99	Cyclo (Leu-pro)	2.3	C_11_H_18_N_2_O_2_	/211.1446	/211.1438	−3.79	70.0657, 138.1276
56	9.03	Chebulagic acid	1.2.3	C_41_H_30_O_27_	953.0897/	953.0871/	−2.73	275.0196, 300.9988, 463.0520
57	9.25	1,2,3,6-Tetra-O-Galloyl-Β-d-Glucose	2.3	C_34_H_28_O_22_	787.0994/	787.1000/	0.76	169.0135, 617.0789, 635.0903
58	9.32	Taxifolin	1.2.3	C_15_H_12_O_7_	/305.0661	/305.0655	−1.97	149.0241, 153.0187
59	9.89	Luteolin-4′-O-Glucoside	1.2.3	C_21_H_20_O_11_	/449.1084	/449.1077	−1.56	153.0181, 287.0545
60	9.95	Loliolide	2.3	C_11_H_16_O_3_	/197.1178	/197.1175	−1.52	133.1012, 179.1066
61	10.02	Myricetin-3-O-Galactoside	1.2.3	C_21_H_20_O_13_	479.0826/	479.0838/	2.50	271.0250, 316.0224
62	10.03	Coniferylaldehyde	2.3	C_10_H_10_O_3_	/179.0708	/179.0705	−1.68	119.0492, 147.0439, 161.0596
63	10.13	1,2,3,4,6-Pentagalloylglucose	2.3	C_41_H_32_O_26_	939.1104/	939.1097/	−0.75	169.0135, 617.1779, 769.0889
64	10.74	Chebulinic acid	1.2.3	C_41_H_32_O_27_	955.1052/	955.1044/	−0.84	169.0135, 337.0201, 465.0669, 785.0832
65	10.80	Quercetin-3Β-D-Glucoside	1.2.3	C_21_H_20_O_12_	463.0876/	463.0883/	1.51	271.0247, 299.9913
66	10.91	Methyl trans-cinnamic acid	1.2.3	C_10_H_10_O_2_	/163.0759	/163.0753	−3.68	103.0545, 131.0491
67	10.92	Isorhamnetin 3-glucuronide	1.2.3	C_22_H_20_O_13_	491.0826/	491.0831/	1.02	297.9753, 312.9990, 328.0223
68	10.98	Vitexin	1.2.3	C_21_H_20_O_10_	/433.1135	/433.1129	−1.39	283.0601, 313.0705
69	11.15	Lariciresinol 4-O-Glucoside	1.2.3	C_26_H_34_O_11_	521.2023/	521.2032/	1.73	329.1391, 341.1400, 491.1926
70	11.39	Isoquercitrin	1.2.3	C_21_H_20_O_12_	/465.1033	/465.1032	−0.22	85.0288, 97.0287, 303.0495
71	11.40	Rutin	1.2.3	C_27_H_30_O_16_	609.1456/	609.1461/	0.82	271.0247, 300.0273
72	11.56	1-O-Trans-cinnamoyl-Beta-d-Glucopyranose	1.2.3	C_15_H_18_O_7_	/311.1131	/311.1121	−3.21	131.0495
73	11.58	Ellagic acid	1.2.3	C_14_H_6_O_8_	300.9984/	300.9989/	1.66	185.0239, 229.0143, 283.9961
74	11.88	Triethyl phosphate	1.2.3	C_6_H_15_O_4_P	/183.0786	/183.0783	−1.64	127.0154, 155.0466
75	12.24	Myricetin	2.3	C_15_H_10_O_8_	/319.0454	/319.0450	−1.25	165.0180, 273.0387, 301.0346
76	12.62	Kaempferol-3-O-Rutinoside	1.2.3	C_27_H_30_O_15_	593.1506/	593.1523/	2.87	284.0324, 285.0403
77	13.44	6-O-[(2E)-3-phenyl-2-propenoyl]-1-O-(3,4,5-trihydroxybenzoyl)-Β-d-Glucopyranose	1.2.3	C_22_H_22_O_11_	461.1084/	461.1091/	1.52	123.0079, 169.0136
78	13.54	(±)-abscisic acid	1.2.3	C_15_H_20_O_4_	/265.1440	/265.1432	−3.02	163.0761, 201.1278, 219.1389
79	13.73	Afzelin	1.2.3	C_21_H_20_O_10_	431.0978/	431.0991/	3.02	255.0297, 285.0404
80	13.73	Kaempferol	1.2.3	C_15_H_10_O_6_	/287.0556	/287.0552	−1.39	153.0181
81	14.04	Matairesinol	2.3	C_20_H_22_O_6_	/359.1494	/359.1495	0.28	114.0915, 137.0597
82	14.15	Quercetin	1.2.3	C_15_H_10_O_7_	301.0349/	301.0353/	1.33	151.0033, 299.0191
83	14.49	Naringenin	1.2.3	C_15_H_12_O_5_	271.0606/	271.0612/	2.21	65.0229, 107.0128, 119.0493
84	15.48	1,6-Bis-O-Galloyl-Beta-d-Glucose	2.3	C_20_H_20_O_14_	483.0775/	483.0781/	1.24	169.0135, 211.0243, 271.0458
85	17.19	Beta-d-Glucopyranose	2.3	C_36_H_58_O_10_	649.3952/	649.3940/	−1.85	487.3432, 469.3330
86	17.19	Glycyrrhetinic acid	1.2.3	C_30_H_46_O_4_	/471.3474	/471.3472	−0.42	107.0858, 187.1479
87	17.70	(10E,15Z)-9,12,13-trihydroxyoctadeca-10,15-dienoic acid	2.3	C_18_H_32_O_5_	327.2172/	327.2177/	1.53	171.1021, 211.1335
88	17.94	Pinocembrin	2.3	C_15_H_12_O_4_	255.0657/	255.0664/	2.74	151.0030, 213.0557
89	18.12	Corchorifatty acid	1.2.3	C_18_H_32_O_5_	327.2172/	327.2178/	1.83	171.1022, 211.1336
90	18.20	Arjungenin	1.2.3	C_30_H_48_O_6_	503.3373/	503.3374/	0.20	409.3116, 503.3383
91	18.37	Asiatic acid	2.3	C_30_H_48_O_5_	/489.3580	/489.3574	−1.23	205.1596, 407.3302, 453.3362
92	18.54	Hydroxybenzaldehyde	2.3	C_7_H_6_O_2_	/123.0446	/123.0443	−2.44	95.0496
93	18.85	(Z)-9,12,13-trihydroxyoctadec-15-enoic acid	2.3	C_18_H_34_O_5_	329.2328/	329.2337/	2.73	127.1119, 139.1118, 171.1018
94	19.55	Aurantiamide acetate	1.2.3	C_27_H_28_N_2_O_4_	/445.2127	/445.2122	−1.12	105.0337, 117.0699, 224.1067
95	20.12	6,8-Dihydroxy-2,2,4,4-Tetramethyl-5-(2-Methylpropanoyl)-9-Propan-2-Yl-9H-Xanthene-1,3-Dione	1.2.3	C_24_H_30_O_6_	/415.2121	/415.2109	−2.89	107.0856, 415.2136
96	21.42	Asperphenamate	1.2.3	C_32_H_30_N_2_O_4_	/507.2284	/507.2277	−1.38	105.0339, 238.1226
97	21.46	Poricoic acid G	1.2.3	C_30_H_46_O_5_	/487.3424	/487.3420	−0.82	95.0860, 203.1794
98	21.47	(Hydroxymethyl)-1,2,6a,6b,9,12a-Hexamethyl-2,3,4,5,6,6a,7,8,8a,10,11,12,13,14b-Tetradecahydro-1h-picene-4a-Carboxylic acid	1.2.3	C_30_H_48_O_5_	487.3423/	487.3425/	0.41	409.3121, 421.3111
99	22.89	Maslinic acid	1.2.3	C_30_H_48_O_4_	/473.3631	/473.3628	−0.63	203.1793, 205.1585
100	23.50	Linolenic acid	1.2.3	C_18_H_30_O_2_	/279.2324	/279.2321	−1.07	81.0705, 95.0860
101	23.81	Oleamide	2.3	C_18_H_35_NO	/282.2797	/282.2792	−1.77	111.1169, 142.1222
102	26.04	Oleanolic acid	1.2.3	C_30_H_48_O_3_	455.3525/	455.3531/	1.32	455.3530
103	26.06	Ursolic acid	1.2.3	C_30_H_48_O_3_	/457.3682	/457.3668	−3.06	191.1795
104	26.43	Hexadecanamide	2.3	C_16_H_33_NO	/256.2640	/256.2633	−2.73	85.1012
105	26.67	Linoleic acid	1.2.3	C_18_H_32_O_2_	279.2324/	279.2328/	1.50	279.2328
106	27.42	Oleic acid	1.2.3	C_18_H_34_O_2_	281.2481/	281.2485/	1.42	281.2485

Origin 1 is U-0.5 h; Origin 2 is R-2 h; Origin 3 is R-4 h.

### Effects of *Triphala* on Physiological Growth

There was no significant difference in animal weights between groups (Supplementary Table S2). Treatments yielded no adverse reactions, including nausea, vomiting, and loss of appetite. The dosages chosen had no significant impact on physiological growth and had no obvious toxic side effects.

### Effects of *Triphala* on Liver Index

**FIGURE 3:**
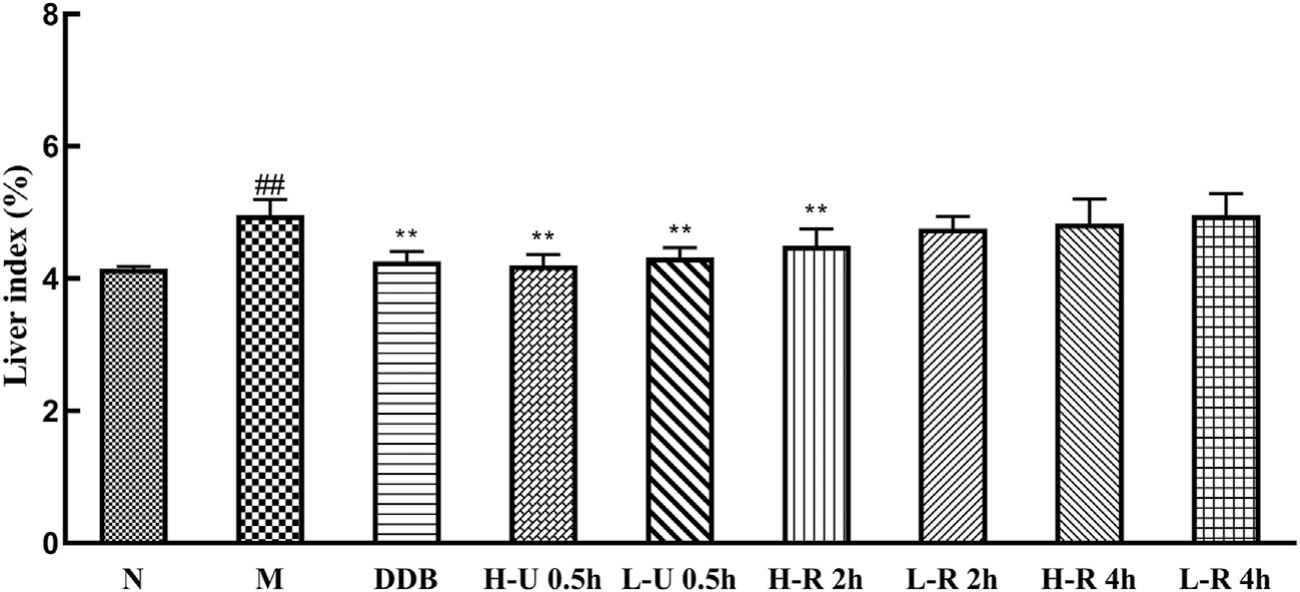
Comparison of mouse liver index ($\bar{x}$ ± s, *n* = 6). Compared with normal control group (N), ^#^ *p* < 0.05, ^##^ *p* < 0.01; compared with model control group (M), *p < 0.05, **p < 0.01.

The liver index is an important indicator of pathological changes caused by liver injury (Figure 3). Compared with the N group, the model groups’ liver index increased significantly (*p <* 0.01), indicating that hepatotoxicity was successfully modeled. Compared with the M group, the liver index of the treatment group was reduced. Group DDB and U-0.5 h differed significantly (*p <* 0.01), and the high dose R-2 h also differed significantly (*p <* 0.01). The results show that all *Triphala* extracts alleviated liver damage in mice, but the ultrasonic extraction method was most effective.

### Effects of *Triphala* on Serum ALT and AST Levels

**FIGURE 4:**
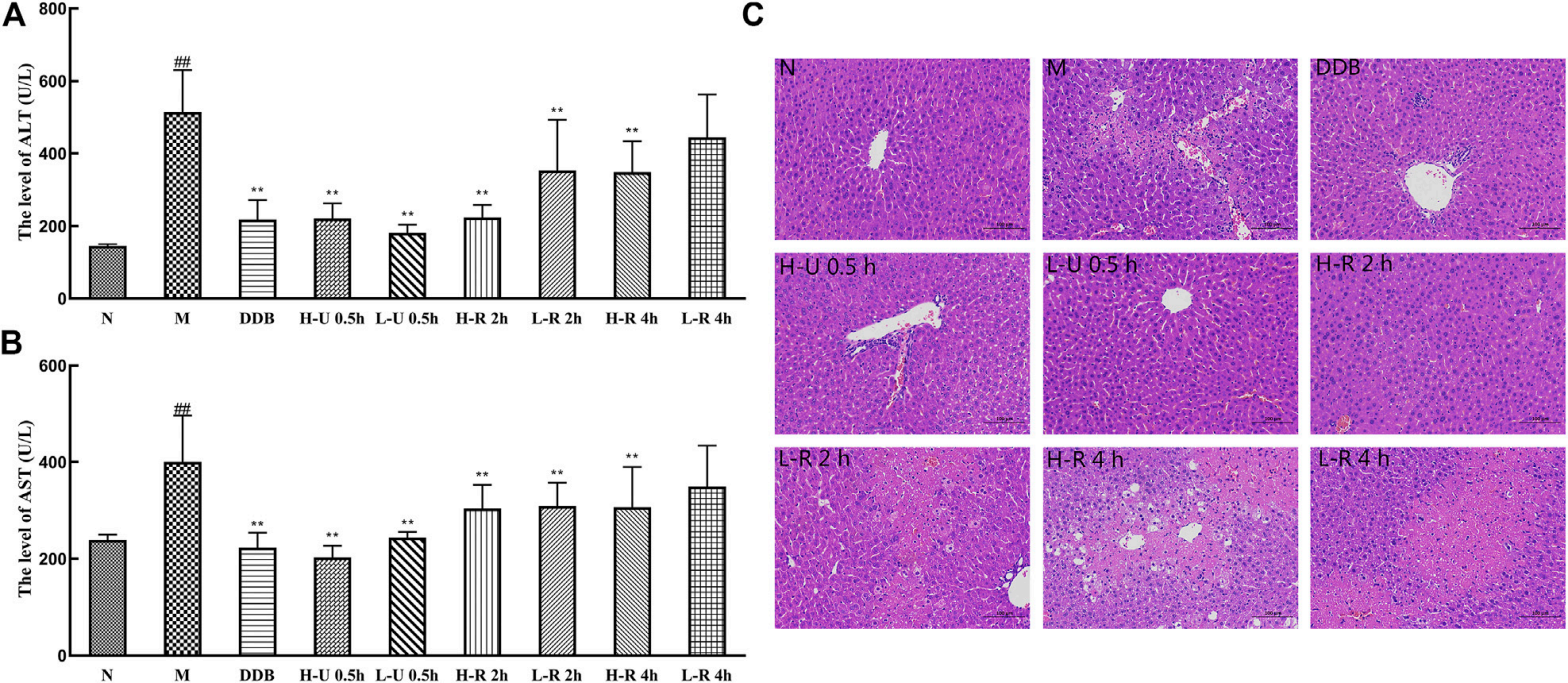
Comparison of serum levels of ALT **(A)** and AST **(B)** ($\bar{x}$ ± s, *n* = 6). Compared with group N, ^#^ *p* < 0.05, ^##^ *p* < 0.01; compared with group M, *p < 0.05, **p < 0.01. Hepatic tissue sections under microscope **(C)** (H&E Stain, × 200).

Compared with the N group, ALT and AST levels in group M were significantly increased (*p <* 0.01; Figure 4A,B). In contrast, all mice treated with U-0.5, R-2, and the high dose R-4 h exhibited significantly reduced ALT and AST (*p <* 0.01). However, mice treated with a low dose of the 4-h reflux extract showed a slight but insignificant decrease in enzyme activity (*p* > 0.05).

### Histopathology

As in Figure 4C, in the normal group, the hepatic lobules were clear in structure, the cords were neatly arranged, and the hepatocytes were rich in cytoplasm and normal in morphology. In the model group, the hepatic lobule structure was destroyed, most hepatocytes were swollen, many hepatocytes were steatotic and necrotic, and many inflammatory cells were infiltrated. In contrast, the liver tissues of the DDB group were nearly normalized; the degree of the hepatocellular lesion was relatively mild. In the U-0.5 h treatment group, the dose dependence was not significant, the hepatic lobule structure was clear, the hepatocyte cytoplasm was abundant, the morphological structure was normal, and a small amount of hepatocyte necrosis, nuclear fragmentation, or dissolution was seen at the edge of the local tissue. In the R-2 h treatment group, the dose dependence was significant. In the high-dose group, the hepatic lobule structure was clear, more hepatocytes showed mild degeneration, and smaller round vacuoles were seen in the cytoplasm. In the low-dose group, a large amount of hepatocyte necrosis, nuclear fragmentation, and dissolution was seen around the central vein and the junction area, and a small amount of hepatocyte balloon-like degeneration was seen at the edge of the necrotic focus. The cells were swollen, the nuclei were centered, and the cytoplasm was vacuolated. In the R-4 h treatment group, the degree of liver cell damage was similar to the model group. The hepatic lobule structure was destroyed, and many inflammatory cells were infiltrated.

### Effects of *Triphala* on Lipid Peroxidation

**FIGURE 5:**
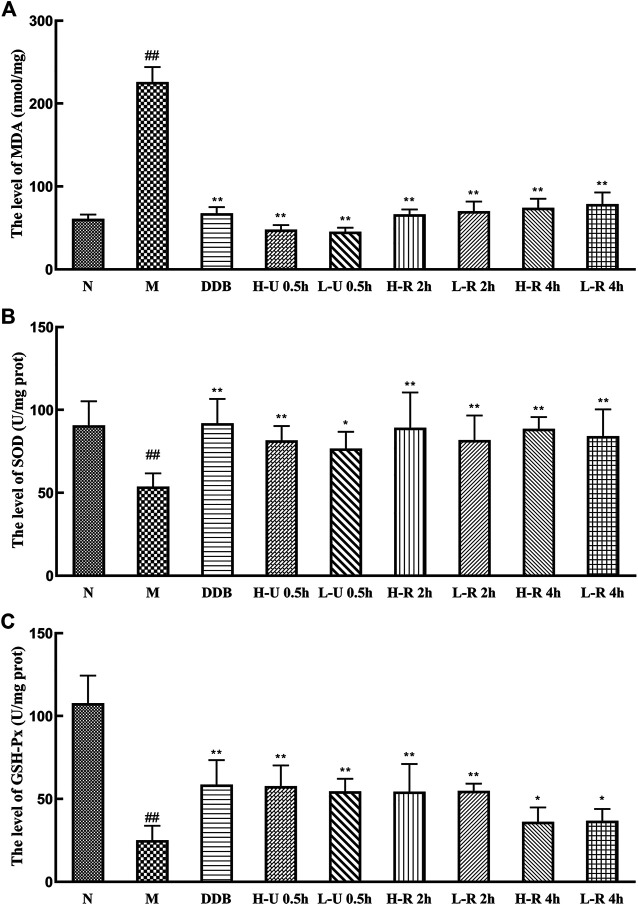
Comparison of liver levels of MDA **(A)**, SOD **(B)** and GSH-Px **(C)** ($\bar{x}$ ± s, *n* = 6). Compared with group N, ^#^ *p* < 0.05, ^##^ *p* < 0.01; compared with group M, *p < 0.05, **p < 0.01.

Compared with the N group, MDA levels in the model group were significantly increased. The levels of SOD and GSH-Px were significantly reduced (*p <* 0.01; Figure 5), indicating that the liver tissue experienced intense oxidative stress and mounted a lipid peroxidation response. Compared with the M group, MDA levels in the different treatment groups were significantly reduced (*p <* 0.01), the levels of SOD were significantly increased (*p <* 0.01), and the levels of GSH-Px were also significantly increased (*p <* 0.05 or 0.01). Thus, we conclude that *Triphala* improved the antioxidant response, relieving liver damage caused by CCl_4_.

### Effects of *Triphala* on Anti-inflammatory Markers

**FIGURE 6:**
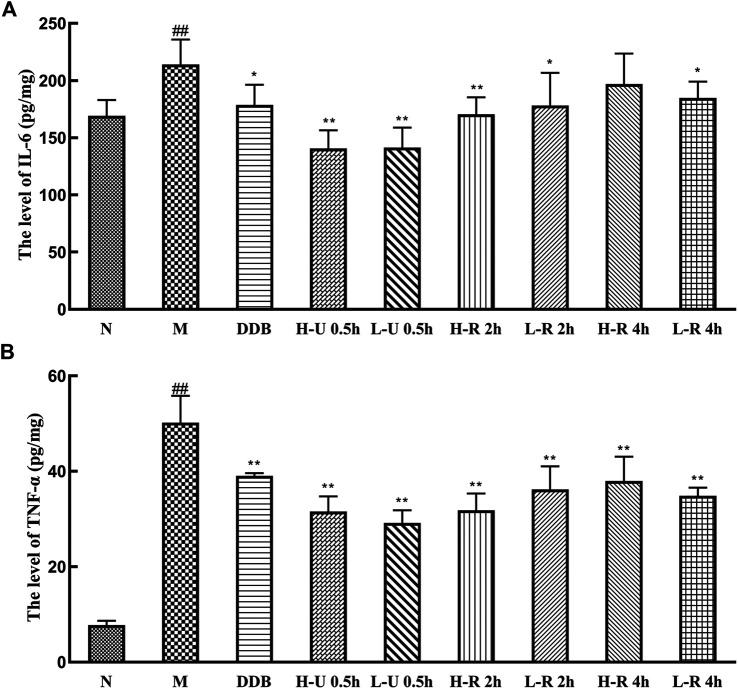
Comparison of liver levels of IL-6 **(A)**, TNF-α **(B)** ($\bar{x}$ ± s, *n* = 6). Compared with group N, ^#^ *p* < 0.05, ^##^ *p* < 0.01; compared with group M, *p < 0.05, **p < 0.01.

Compared with the N group, IL-6 and TNF-α levels in the model group were significantly increased (*p <* 0.01; Figure 6). Compared with the M group, the levels of TNF-α were significantly reduced in the treatment groups (*p <* 0.01). IL-6 levels were significantly reduced in the U-0.5 and H-R-2 h groups (*p <* 0.01) and significantly differed in the L-R-2 and R-4 h groups (*p <* 0.05). Thus, the ultrasonic extract of *Triphala* provided a greater anti-inflammatory effect.

### Effect of *Triphala* on Nrf-2, HO-1 and NQO-1 Genes and Protein Expression

**FIGURE 7:**
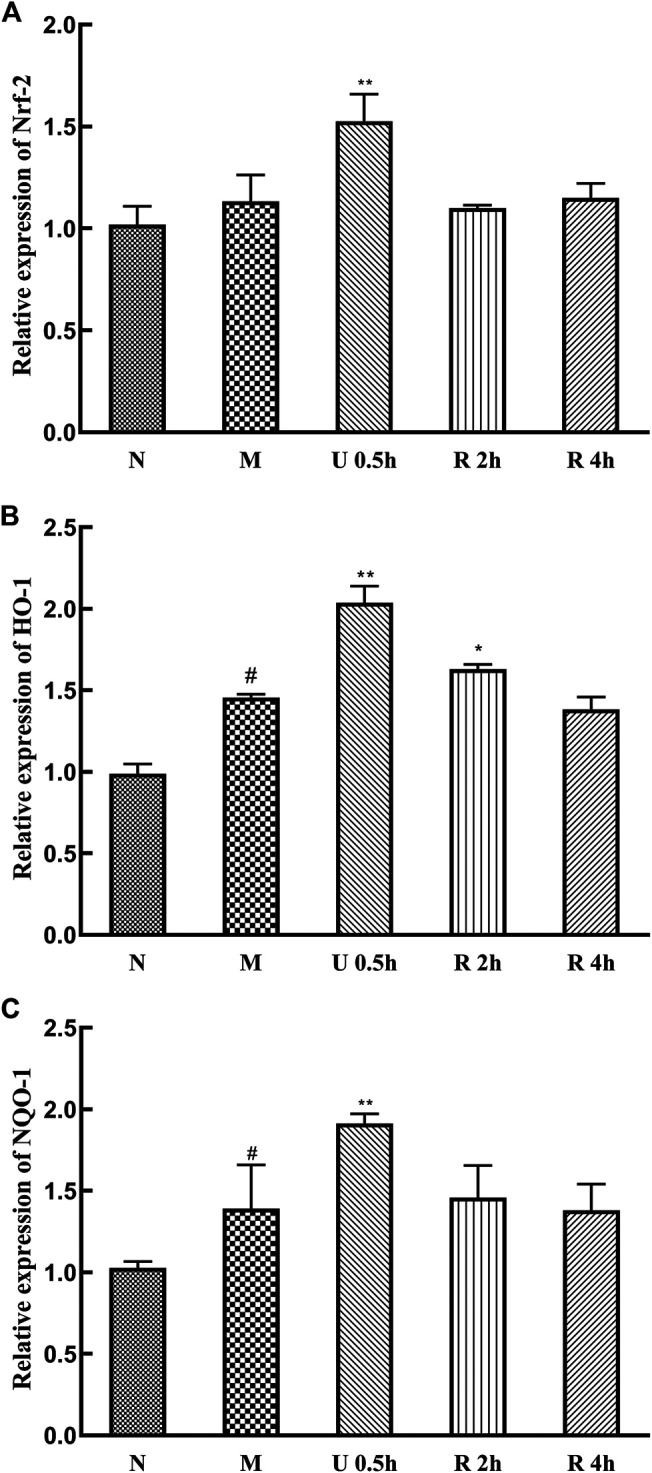
Relative expression of Nrf-2 **(A)**, HO-1 **(B)** and NQO-1 **(C)** mRNA ($\bar{x}$ ± s, *n* = 6). Compared with group N, ^#^ *p* < 0.05, ^##^ *p* < 0.01; compared with group M, *p < 0.05, **p < 0.01.

The expression levels of Nrf-2, HO-1, and NQO-1 mRNA in the nuclei of liver tissues in the treatment groups were significantly increased in the treatment groups (Figure 7). Specifically, Nrf-2, HO-1, and NQO-1 mRNA expression was highest in the group treated with the ultrasonic *Triphala* extract (*p <* 0.01). CCl_4_ also promotes the expression of these genes.

**FIGURE 8:**
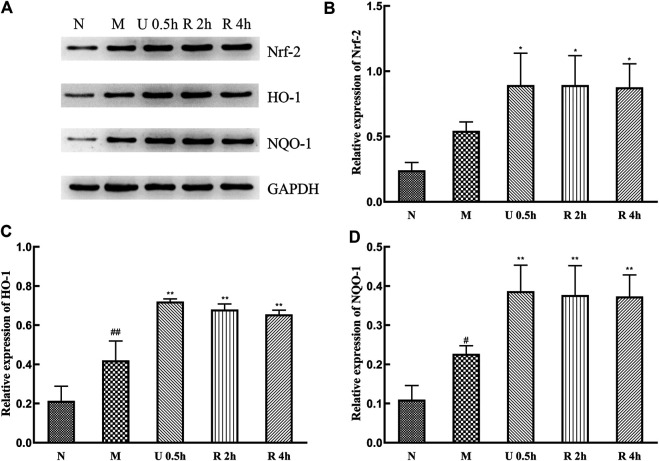
Western blot detection of Nrf-2 signaling pathway and related proteins **(A)**, Relative expression of Nrf-2 **(B)**, HO-1 **(C)** and NQO-1 **(D)** protein ($\bar{x}$ ± s, *n* = 6). Compared with group N, ^#^ *p* < 0.05, ^##^ *p* < 0.01; compared with group M, *p < 0.05, **p < 0.01.

Expression levels of Nrf-2, HO-1, and NQO-1 protein in the nuclei of liver tissues significantly increased in the treatment groups (Figure 8), with no substantial difference between extraction methods. CCl_4_ also promotes the expression of these proteins.

## Conclusion and Discussion

Liver disease carries high morbidity and mortality worldwide. Reactive oxygen species (ROS) play a pivotal role in the occurrence and development of chronic liver disease (Kuriakose et al., 2017). Plants are rich in antioxidant ingredients, which are important in reducing the pathogenesis of oxidative stress due to their free radical scavenging effect (Firuzi et al., 2011). Nevertheless, before plants are used as therapies in modern medical systems, they need to be systematically verified and screened. *Triphala* has been used as a traditional Ayurvedic medicine for centuries and has been shown to have great potential to promote antioxidant activity (Prasad and Srivastava, 2020). The compound is known to scavenge free radicals, restore antioxidant enzyme and non-enzyme levels, reduces lipid peroxidation, and has good therapeutic prospects for liver diseases. As the main active ingredient of *Triphala*, tannin is also the main substance of antioxidant activity.

CCl4 is widely used in the preparation of liver injury models and is one of the commonly used chemical drugs to verify the hepatoprotective activity of plant-based drugs. The oxidative damage caused by CCl_4_ is a good model for screening anti-plant drugs for liver protection activity (Nada et al., 2010). Free radicals (∙CCl3) are the active metabolites of CCl_4_, mainly related to liver damage caused by CCl_4_ (Wu et al., 2007), reacting with oxygen to form trichloromethyl peroxide radical (CCl_3_OO∙), which initiates a chain reaction of lipid peroxidation and attacks and destroys polyunsaturated fatty acids, especially those related to phospholipids (Szymonik-Lesiuk et al., 2003; Ranawat et al., 2010).

This study examined three Triphala extracts for protective activity with DDB as a positive control drug. The results show that the three tested extracts have a protective effect on damaged liver cells, but the most protective was the ultrasonic extraction, which significantly reduced serum ALT and AST and MDA in liver tissues increased SOD and GSH-Px activities. These results show that *Triphala* can better improve free radical scavenging and reduce cell damage caused by free radicals. In addition, CCl_4_-induced oxygen free radicals can produce Kupffer cells, which mediate the liver inflammatory response by inducing TNF-α and interleukin (Yu et al., 2014). Current research shows that *Triphala* can reduce the overexpression of TNF-α and IL-6 in CCl_4_-induced mouse liver tissues, inhibit inflammation, and provide a hepatoprotective effect.

The Nrf-2 signaling pathway is one of the body’s most important signaling pathways to cope with oxidative stress injury. It can increase the antioxidant level by up-regulating antioxidant proteins in liver cells (Li et al., 2015). Under normal conditions, Nrf-2 is in a state of inhibition. When free radicals attack the body, Nrf-2 enters the nucleus and activates heme oxygenase-1 (HO-1) and phosphoramidite adenine dinucleotide quinone oxidoreductase-1 (NQO-1), further catalyzing heme degradation and eliminating free radicals from the body (Hseu et al., 2012). This study has shown that *Triphala* can significantly increase both transcript and protein expression of Nrf-2, HO-1, and NQO-1 in damaged liver tissues, regulates the Nrf-2 signaling pathway, and improves the performance of the body’s antioxidant system.

In summary, the three tested *Triphala* extracts have a hepatoprotective effect, but there were clear differences in efficacy between preparations. The ultrasonic preparation of *Triphala* was most effective, suggesting that macromolecular substances mediate the protective effect against liver injury, and the loss of macromolecular substances to hydrolysis reduces hepatoprotective potency. During the hydrolysis of *Triphala*, small molecules such as gallic and ellagic acid increase, but studies have found that gallic acid and ellagic acid exhibit poor absorption, low bioavailability, and easy saturation. When the content is saturated, the increase in content has little effect on the efficacy (Seeram et al., 2004; Ahmed et al., 2018). Structure-activity analysis has shown a large number of phenolic hydroxyl groups in the molecular structure of chebulagic and chebulinic acid, suggesting significant antioxidant activity. Therefore, treatment with botanical medicines rich in hydrolyzed tannin must be prepared in a way that optimizes efficacy. There are various preparation processes, but extraction methods should be controlled to meet the consistency requirements for the biological activities of medications. This study’s most significant finding is that low-temperature extraction is essential to retaining bioactive hydrolyzed tannins and improving *Triphala’s* hepatoprotective efficacy.

## Data Availability

The raw data supporting the conclusions of this article will be made available by the authors, without undue reservation, to any qualified researcher.
