# Supplementary material for: Hepatoprotective Effects of Different Extracts From Triphala Against CCl4-Induced Acute Liver Injury in Mice
Source: Front Pharmacol. 2021 Jul 5;12:664607. doi: 10.3389/fphar.2021.664607 (PMC8287969; doi:10.3389/fphar.2021.664607)
Supplement: Supplementary file 1 [file datasheet1.docx]

Supplementary Material

**Table S1.** **Primer sequences for RT-PCR**

| Gene | Sequences（5'-3') | Annealing  temp (°C) | Product size  (bp) |
| --- | --- | --- | --- |
| M-Actin | F: GTGCTATGTTGCTCTAGACTTCG  R: ATGCCACAGGATTCCATACC | 60 | 174 |
| M-Nrf-2 | F: CAGCCATGACTGATTTAAGCAG  R: CAGCTGCTTGTTTTCGGTATTA | 60 | 107 |
| M-NQO-1 | F: TGGGAGGAGACCCCACTCTA  R: GGAATGGACTTGCCCAGGTG | 60 | 149 |
| M-HO-1 | F: TCCTTGTACCATATCTACACGG  R: GAGACGCTTTACATAGTGCTGT | 60 | 198 |

**Table S2.** **Comparison of body weight of mice (**$\bar{\boldsymbol{x}}$**±s，n=6)**

| Group | First day weight/g | Third day weight/g | Fifth day weight/g | Seventh day weight/g |
| --- | --- | --- | --- | --- |
| N  M  DDB  L-U-0.5 h  H-U-0.5 h  L-R-2 h  H-R-2 h  L-R-4 h  H-R-4 h | 25.72±1.56  25.01±1.20  26.28±2.62  24.77±0.89  26.32±1.36  27.29±2.06  27.68±1.98  28.14±1.42  28.74±1.46 | 29.36±2.59  30.72±2.20  30.13±1.13  30.17±1.26  29.02±0.80  28.86±1.74  29.38±2.12  30.95±2.31  30.49±1.99 | 30.33±1.32  30.14±2.48  29.64±2.31  29.77±2.32  30.98±1.49  30.03±2.55  30.65±2.87  31.41±2.91  31.51±2.96 | 31.19±1.76  30.35±2.01  31.50±2.28  31.14±1.50  31.35±1.97  31.40±2.22  31.16±3.42  31.96±2.69  31.07±3.29 |

Note: Compared with group N, ^#^P<0.05, ^##^P<0.01; compared with group M, *P<0.05, **P<0.01.
